# A multiphasic approach to solve misidentification of *Cutibacterium acnes* as *Atopobium vaginae* during routine bacterial screening of platelet concentrates using the VITEK 2 system

**DOI:** 10.1099/acmi.0.000539.v3

**Published:** 2023-06-16

**Authors:** Dilini Kumaran, Carmelie Laflamme, Sandra Ramirez-Arcos

**Affiliations:** ^1^​ Canadian Blood Services, Ottawa, Ontario, Canada; ^2^​ University of Ottawa, Ottawa, Ontario, Canada

**Keywords:** *Atopobium vaginae*, *Cutibacterium acnes*, VITEK 2, MALDI-TOF, misidentification, multiphasic identification, 16S RNA

## Abstract

Skin flora bacteria, such as *Cutibacterium acnes *, are the predominant contaminants of blood products used for transfusion. Platelet concentrates (PCs), a therapeutic product used to treat patients with platelet deficiencies, are stored at ambient temperature under agitation, providing ideal conditions for bacterial proliferation. At Canadian Blood Services, PCs are screened for microbial contamination using the automated BACT/ALERT culture system. Positive cultures are processed and contaminating organisms are identified using the VITEK 2 system. Over a period of approximately 2 years, several PC isolates were identified as *Atopobium vaginae* to a high level of confidence. However, since *A. vaginae* is associated with bacterial vaginosis and is not a common PC contaminant, a retrospective investigation revealed that in all cases *C. acnes* was misidentified as *A. vaginae *. Our investigation demonstrated that the media type used to grow PC bacterial isolates can have a significant impact on the results obtained on the VITEK 2 system. Furthermore, other identification methods such as matrix-assisted laser desorption/ionization time-of-flight mass spectrometry (MALD-TOF MS) and PCR amplification of the 16S RNA gene were only partially successful in the identification of *C. acnes *. Therefore, our findings support a multiphasic approach when PC isolates are identified as *A. vaginae* by the VITEK 2 system for proper identification of *C. acnes* using macroscopic, microscopic and other biochemical analyses.

## Data Summary

The data generated in this study are summarized in Table 1 and include the results of bacterial identification using different approaches as described in the Methods section. No supporting external data were generated in this study.

**Table 1.** Identification of PC isolates using the VITEK 2 system when grown on different media

Lab ID	Initial ID	VITEK results			Other identification methods			
		Media type			MALDI-TOF†	16S rRNA PCR‡	Rapid ID 32A§	API 20A¶
		CBA	Oxy	TSAb	nt	nt	nt	nt
19 482	* A. vaginae *	C. acnes*	nt	* A. vaginae* (EI)	nt	nt	nt	nt
20 047	* A. vaginae *	C. acnes*	* A. vaginae* (EI)	nt	* C. acnes *	nt	nt	nt
21 101	* A. vaginae *	* C. acnes* (EI)	* A. vaginae* (VI)	nt	NI	Negative	* C. acnes *	nt
21 111	* A. vaginae *	* A. vaginae* (VI)	* A. vaginae* (VI)	nt	nt	nt	nt	nt
21 112	* A. vaginae *	* A. vaginae* (VI)	* A. vaginae* (VI)	nt	nt	nt	nt	nt
21 090	* A. vaginae *	* C. acnes* (EI)	C. acnes* (VI)	* C. acnes* (EI)	NI	Negative	* C. acnes *	nt
21 261	* A. vaginae *	* A. vaginae* (EI)	nt	* A. vaginae* (EI)	NI	Negative	NI	*C. acnes/C. granulosum*
21 266	* A. vaginae *	* A. vaginae* (EI)	nt	* A. vaginae* (EI)	* C. acnes *	nt	nt	nt
21 224	* C. acnes *	* C. acnes* (EI)	* A. vaginae* (VI)	* C. acnes* (EI)	NI	Negative	* C. acnes *	nt
21 168	* C. acnes *	* A. vaginae* (EI)	*nt*	C. acnes*	* C. acnes *	nt	nt	nt
21 202	* C. acnes *	C. acnes*	* C. acnes* (VI)	nt	* C. acnes *	nt	nt	nt
21 201	* C. acnes *	C. acnes*	C. acnes*	nt	nt	nt	nt	nt
21 255	* C. acnes *	* C. acnes* (EI)	C. acnes*	nt	nt	nt	nt	nt
21 292	* C. acnes *	* C. acnes* (EI)	C. acnes*	nt	nt	nt	nt	nt
21 216	* C. acnes *	* C. acnes* (EI)	C. acnes*	* C. acnes* (EI)	nt	nt	nt	nt
21 225	* C. acnes *	* C. acnes* (EI)	C. acnes*	* C. acnes* (EI)	nt	nt	nt	nt
21 220	* C. acnes *	* C. acnes* (EI)	C. acnes*	* C. acnes* (EI)	nt	nt	nt	nt
21 193	* C. acnes *	* C. acnes* (VI)	nt	C. acnes*	nt	nt	nt	nt
21 263	* C. acnes *	* C. acnes* (EI)	nt	C. acnes*	nt	nt	nt	nt
21 162	* C. acnes *	C. acnes*	nt	C. acnes*	nt	nt	nt	nt
21 169	* C. acnes *	C. acnes*	nt	C. acnes*	nt	nt	nt	nt
* C. sordellii* ATCC 9714	nt	nt	nt	nt	nt	nt	* C. sordellii *	* C. sordellii *
* C. acnes* ATCC 6919	na	* C. acnes* (EI)	*C. acnes/C. granulosum*	* C. acnes* (VI)	nt	Positive	nt	nt
* A. vaginae* BAA-55	na	* A. vaginae* (EI)	NI	* A. vaginae* (EI)	* A. vaginae *	Negative	nt	nt

*ID obtained following catalase test prompted by VITEK 2 system.

†Cultures for MALDI-TOF MS were subcultured on CDC media as per the institute’s protocols.

‡Isolates cultured on TSAb prior to PCR.

§Isolates cultured on CBA for RAPID ID 32A testing.

¶Isolates cultured on TSAb for API 20A.

EI, excellent ID (96–99%); NI, not identified; NT, not tested; VI, very good ID (93–95%).

**Impact Statement**

Microbial identification with automated systems based on biochemical reactions is common practice in clinical and industrial settings. These systems provide information about contaminants that aid decision making for patient treatment, or in the case of blood suppliers, provides information for blood product disposition, follow up of blood donors, and in some cases, follow up of transfusion patients. It is therefore very important to have accurate microbial identification results. To our knowledge, we report an issue so far not yet documented in literature concerning the misidentification of the skin flora bacterium *Cutibacterium acnes* as *Atopobium vaginae,* a microorganism involved in bacterial vaginosis. We showed that the automated VITEK identification system misidentified *C. acnes* on several occasions and that the most plausible cause for the unexpected results was the culture media used to isolate the organisms. Recommendations on how to get accurate VITEK identification results when a blood contaminant is unexpectedly identified as *A. vaginae,* include using vendor’s recommended culture media, assessing microscopic and macroscopic morphologies, and applying alternative identification systems, such as manual identification strips, MALDI-TOF MS or molecular methods to confirm VITEK results.

## Introduction

Platelet concentrates (PCs) are manufactured by Canadian Blood Services to treat patients with bleeding disorders or platelet deficiencies. PCs are screened for bacterial contamination using the BACT/ALERT 3D system with aerobic (BPA) and anaerobic (BPN) culture bottles [1]. When a bottle flags positive, the contaminant is isolated by subculturing samples onto different agar types, including tryptic soy agar with 5 % sheep blood (TSAb), chocolate agar and anaerobic agar, such as CDC anaerobic blood agar with 5 % sheep blood (CDC) or OxyPRAS Plus (* Brucella* agar containing 5 % sheep blood and oxyrase) (Oxy), which are then incubated under different environmental conditions (aerobic, capnophilic and anaerobic, respectively). When growth is observed, a Gram stain is performed, and the isolates are identified using the VITEK 2 system and the appropriate VITEK 2 cards chosen based on the algorithm provided by the manufacturer. No other complementary tests are performed unless prompted by the VITEK 2 system. Data gathered at Canadian Blood Services between 2017–2019 indicate that the most isolated bacterial contaminants of PCs are skin commensals such as *Cutibacterium acnes *, and staphylococcal species [1]. *C. acnes,* an anaerobic, aerotolerant, catalase-positive, Gram-positive, rod-shaped bacterium [2], is the most routinely isolated contaminant of PCs, accounting for approximately 70 % of bacterial species identified from positive BACT/ALERT cultures [1]. The VITEK 2 system was adopted for identification of blood product contaminants in 2019, prior to which, microbial identification at Canadian Blood Services was performed with the manual analytical profile index (API) identification system. In the 2 years following implementation of identification with the VITEK 2 system, there were several unprecedented instances of anaerobic bacterial isolates being identified as *Atopobium vaginae* to a high level of confidence (>95 %). Furthermore, several isolates could only be identified following a catalase test prompted by the VITEK 2 system, since it could not differentiate between *A. vaginae* and *C. acnes *.

*A. vaginae,* a member of the vaginal microflora, is a strict anaerobic, Gram-positive, catalase-negative, rod-shaped coccus [3], and is usually associated with bacterial vaginosis [4]. It should be noted, however, that there have been reports of *A. vaginae* being isolated from blood cultures from individuals experiencing transient bacteraemia [5], infective endocarditis [6] and sepsis [7]. Since *A. vaginae* was an unusual organism to be detected during screening of PCs obtained from healthy blood donors, a retrospective study was initiated to determine if these isolates may have been misidentified. The steps taken to ascertain the identities of these isolates have been described herein and provide recommendations on how to proceed when a PC isolate is identified as *A. vaginae *.

## Methods

### Bacterial strains

For the investigation, 8 catalase-positive PC isolates that were misidentified as *A. vaginae* were selected, together with 13 other isolates that were identified as *C. acnes* (see Table 1). The *C. acnes* ATCC 6919 strain and *A. vaginae* ATCC BAA-55 strain were used to evaluate microscopic and macroscopic morphology, and served as controls for VITEK identification and *C. acnes* 16S RNA PCR. *Clostridium sordellii* ATCC 9714 served as the control for API tests. *Staphylococcus aureus* ATCC 25923 and *Streptococcus pyogenes* ATCC 19615 were used as the positive and negative controls, respectively, for the catalase test. *Clostridium septicum* ATCC 12464 and *Bacteriodes ovatus* ATCC BAA-1296, which are quality control (QC) strains for the ANC card recommended by the VITEK 2 vendor, were used to perform weekly qualifications of the VITEK 2 system.

### Agar media

Four agar media were used in this study. Three media, TSAb and CDC or Oxy, were used for the initial isolation of anaerobes during routine PC testing as per standard protocols at Canadian Blood Services. Columbia blood agar supplemented with 5 % sheep blood (CBA) was used to grow the QC controls for the anaerobic (ANC) VITEK 2 card as per the vendor’s recommendations.

### Assessing macroscopic and microscopic colony morphology

The ATCC strains of *C. acnes* (ATCC 6919) and *A. vaginae* (BAA-55) were grown on TSAb, CDC and CBA plates for 72 h, under anaerobiosis at 37 °C, and the colony morphology of the two species was assessed. Furthermore, smears were prepared, and Gram stain was performed to determine the microscopic differences between the two species. Additionally, a catalase test was performed as per the catalase test protocol described by the American Society of Microbiology [8]. Briefly, four well-isolated colonies of the ATCC *C. acnes *, *A. vaginae* and the catalase control strains grown on TSAb were picked using a sterile loop, ensuring that no agar was transferred, and were deposited on a clean, labelled microscope slide, to which a single drop of 3 % hydrogen peroxide was added, after which the slides were assessed for the production of effervescence.

### Impact of different growth media on identification by the VITEK 2 system

All isolates were grown on CBA and Oxy or TSAb and five isolates (21 090, 21 224, 21 216, 21 225 and 21 220) were randomly chosen to be grown on the three media types. Isolates were streaked on agar media and incubated under anaerobic conditions at 37 °C for up to 48 h, until sufficient growth was obtained; isolates derived from each media type were identified with the ANC card using the VITEK 2 system (version 9.01) as per the manufacturer’s instructions, with bacterial suspensions prepared in 0.45 % sterile saline solution in polystyrene tubes, and densities verified using the VITEK 2 DENSICHEK apparatus corresponding to MacFarland 3. The ATCC strains of *A. vaginae* and *C. acnes* served as controls and were grown on the three media types for 72 and 48 h, respectively.

### Identification by alternative methods

Matrix-assisted laser desorption/ionization time-of-flight mass spectrometry (MALD-TOF MS) was performed at the Ottawa Hospital reference clinical microbiology laboratory as per the institution’s protocols. Briefly, colonies sub-cultured on CDC media were spotted on target plates and identified using MALDI Biotyper RTC. Of the eight PC isolates that were initially identified as *A. vaginae *, five were chosen for MALDI-TOF MS analysis: two were chosen because these isolates identified as *A. vaginae* when grown on two of the three media types tested (21 261, 21 266), one identified as *C. acnes* regardless of the media type tested (21090), and two isolates identified as *A. vaginae* or *C. acnes* depending on the media type the suspension was made from (20 047, 21 101) (Table 1). Three additional PC isolates (21 224, 21 168, 21 202) that were initially identified as *C. acnes* were included in the MALDI-TOF MS analysis, along with the ATCC *A. vaginae* isolate that served as a control. Isolates 21 101, 21 090, 21 261 and 21 224 could not be identified using MALDI-TOF MS and were assessed using a primer pair that amplifies a section of the *C. acnes* 16S rRNA gene previously described by Bernard *et al*. [9]. The PCR products were run on a 1.5 % agarose gel and the presence of amplicons was assessed. The API RAPID ID 32A strips were used to identify isolates that could not be identified using either the MALDI-TOF MS or PCR methods. Briefly, isolates were sub-cultured on CBA for 48 h to obtain sufficient growth, and suspensions were prepared and dispensed as per the manufacturer’s instructions. The isolate that could not be identified using the RAPID strip was then identified using the API 20A strip following subculture on TSAb for 48 h to allow for sufficient growth, after which suspensions were prepared and dispensed as per the manufacturer’s instructions.

## Results and discussion

### The microscopic and macroscopic morphology and catalase reaction of *A. vaginae* is distinguishable from that of *C. acnes*

**Fig. 1.**
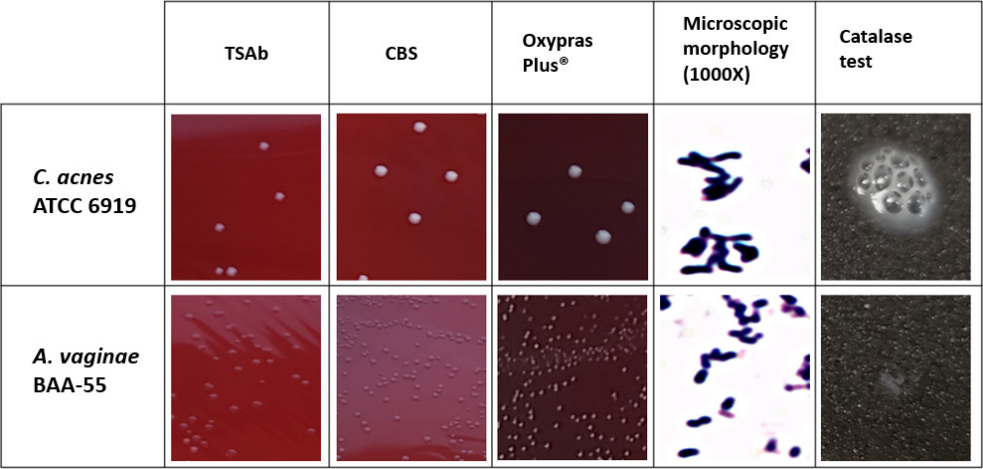
Differences between *C. acnes* and *A. vaginae *. The macroscopic differences observed in *C. acnes* and *A. vaginae* when grown on different agar types under anaerobic conditions. Microscopic morphology and the catalase test results indicating distinguishable characteristics between the two species.

The colony and microscopic characteristics and catalase reaction of *C. acnes* and *A. vaginae* are shown in Fig. 1. *C. acnes* produces small white circular convex colonies with regular margins, while *A. vaginae* produces very small translucent to grey colonies on all three types of media tested. Both species were able to grow more luxuriously on Oxy plates. Microscopically, *C. acnes* is a Gram-positive pleomorphic rod, while *A. vaginae* is a Gram-positive short rod-like coccus. *C. acnes* gives rise to a strong catalase-positive reaction, while *A. vaginae* is catalase-negative.

### Media type affects identification by the VITEK 2 system

The VITEK 2 results obtained for each isolate are listed in Table 1. CDC, Oxy or TSAb were used for initial isolation as per standard procedures implemented at Canadian Blood Services. When isolates were cultured on CBA, TSAb and Oxy for VITEK identification, sufficient growth was obtained more quickly on Oxy plates (as early as 26 h of incubation) compared to the other media. Approximately 76 % of isolates grown on CBA and TSAb were identified as *C. acnes *, while approximately 23 % of isolates were identified as *A. vaginae *. Isolates grown on TSAb were 10 % more likely to require further testing (catalase test) to be able to differentiate between the two species when compared to isolates grown on CBA. On the other hand, approximately 38.5 % of isolates grown on Oxy media were identified as *A. vaginae *. The control ATCC 6919 isolate of *C. acnes* was identified as *C. acnes* when grown on TSAb and CBA, but the system could not differentiate between *C. acnes* and *Cutibacterium granulosum* when grown on Oxy. Similarly, the control *A. vaginae* strain was correctly identified when grown on CBA and TSAb but could not be identified when grown on Oxy. A comparison of the biochemical reactions of isolates that had different IDs depending on the agar medium used indicated that the biochemical profiles only differed in three–five reactions, and these differences varied depending on the isolate tested and the level of discrimination obtained. A few of the reactions that distinguished *A. vaginae* from *C. acnes* include utilization of arginine (ARG), *N*-acetyl-d-glucosamine (NAG), d-ribose 2 (dRIB2), and the Ellman’s (ELLM) and phenylphosphonate (OPS).

### All isolates initially identified as *A. vaginae* were identified as *C. acnes* using a multiphasic approach

Four of the eight samples analysed by MALDI-TOF MS were identified as *C. acnes* (20 047,21 266, 21 168, 21 202). The four isolates that could not be identified were assessed using PCR amplification of a section of the *C. acnes* 16S RNA gene, but none of these four isolates gave rise to an amplicon as observed for the ATCC *C. acnes* isolate. The API Rapid ID 32A strip identified three of these isolates as *C. acnes* (21 101, 21 090, 21 224) while the fourth isolate (21 261) was identified as *C. acnes/C. granulosum* using the API 20A strip (Table 1).

## Conclusion

The VITEK 2 system allows for the rapid and accurate identification of clinically relevant bacterial isolates. Anaerobic bacteria together with *Corynebacterium* species can be identified using the VITEK 2 ANC card. It consists of 36 colorimetric enzymatic reactions, including a combination of biochemical, fermentation, glycosidase and arylamidase tests [10].

Once the card is loaded into the system, the card is assessed for colorimetric changes hourly and the results obtained are matched against an established database. The results of this study demonstrate that the VITEK 2 system can misidentify *C. acnes* isolates as *A. vaginae *. Furthermore, the results indicate that the media used to sub-culture bacterial isolates prior to identification may impact on the ability of the VITEK 2 system to accurately identify isolates.

CBA is the supplier-recommended medium to subculture isolates used for QC testing of the ANC cards. Furthermore, supplier recommendations favour relatively fresh anaerobic cultures (24–48 h) for the preparation of the suspension used for identification. Cultures grown on Oxy can grow more rapidly, giving rise to more mature cultures than those grown on CBA or TSAb over the same period of time, which could lead to skewed enzymatic profiles in the ANC card, resulting in the higher proportion of misidentifications. The results obtained in our study suggest that it may be preferable to use CBA for subculture prior to identification, even if Oxy media may appear to be better at promoting the primary growth and isolation of anaerobic bacteria.

Interestingly, a study conducted by Cassity recommended using Oxy plates for identification with VITEK since that media promotes growth of anaerobic bacteria [11]; however, in that study, bacterial identification results were not evaluated. Furthermore, our results highlight the limitations of methods such as MALDI-TOF MS and 16S rRNA amplification (in the absence of sequencing using universal primers [12]) to be able to identify certain isolates. This could potentially be caused by limited databases for reference for MALDI-TOF MS, and primer pair mismatches during PCR amplification in the absence of whole-genome sequences.

Overall, our results illustrate the challenges posed by automated identification of fastidious or slow-growing bacteria such as *C. acnes *. Misidentification of bacteria, especially in clinical settings, could have serious repercussions for patient treatment. Based on our findings, we recommend having a multiphasic approach for the identification of *C. acnes* when initial identification with the VITEK system results in a Gram-positive anaerobic bacterium such as *A. vaginae *, particularly, if it is a bacterium isolated from contaminated transfusable blood components. It is important to consider micro- and macroscopic morphologies, catalase reaction and the use of alternative methods such as MALDI-TOF, if available, or manual identification strips.
